# Genomic Insights Into Convergent Evolution: Adaptation to Rocky Habitats in Rock-Inhabiting Fungi

**DOI:** 10.1093/molbev/msaf249

**Published:** 2025-10-06

**Authors:** Rong Fu, Luwen Yan, Shunxian Wang, Dongsheng Wei, Qi Wu, Xingzhong Liu, Meichun Xiang

**Affiliations:** College of Bioengineering and Technical, Tianshui Normal University, Tianshui, China; State Key Laboratory of Microbial Diversity and Innovative Utilization, Institute of Microbiology, Chinese Academy of Sciences, Beijing, China; College of Life Science, University of Chinese Academy of Sciences, Beijing, China; Department of Microbiology, College of Life Science, Nankai University, Tianjin, China; Department of Microbiology, College of Life Science, Nankai University, Tianjin, China; Department of Microbiology, College of Life Science, Nankai University, Tianjin, China; State Key Laboratory of Microbial Diversity and Innovative Utilization, Institute of Microbiology, Chinese Academy of Sciences, Beijing, China; Department of Microbiology, College of Life Science, Nankai University, Tianjin, China; State Key Laboratory of Microbial Diversity and Innovative Utilization, Institute of Microbiology, Chinese Academy of Sciences, Beijing, China; College of Life Science, University of Chinese Academy of Sciences, Beijing, China

**Keywords:** rock-inhabiting fungi, genomic features, convergent evolution, mannosyltransferase, meristematic growth

## Abstract

Rock-inhabiting fungi (RIF), obligate colonizers of bare rocks, are primarily distributed across two major phylogenetic classes: Dothideomycetes and Eurotiomycetes. These fungi display striking convergence in morphology and physiology, characterized by meristematic growth, melanized cell walls, and extreme stress tolerance. However, the genomic underpinnings of this adaptive convergence remain poorly understood. Here, through comparative genomic analysis of 9 RIF and 18 non-RIF fungi, we revealed that RIF possess compact, gene-dense genomes marked by contraction of genes involved in nutrient uptake and secondary metabolism, alongside expansions in cell wall biosynthesis, lipid metabolism, and stress-responsive pathways. We identified two genes under positive selection across multiple RIF lineages: Ino80 ATPase (chromatin remodeling) and the ER chaperone BiP (protein folding). Further evidence of convergence was found in the mannosyltransferase *Mnn9*, a key enzyme in cell wall assembly, where two RIF-specific amino acid substitutions were predicted to enhance protein stability. Additionally, a unique *Mnn9*-like clade has expanded exclusively in RIF. RNAi-mediated knockdown of an *Mnn9*-like gene in *Rachicladosporium* sp. confirmed its role in cell wall mannosylation, osmotic stress response, and the transition from meristematic to filamentous growth. Our findings elucidate a set of common genomic adaptations and highlight the specialized evolution of the *Mnn9* family in driving the convergent success of phylogenetically diverse RIF in rocky environments.

## Graphical Abstract

**Graphical Abstract:**
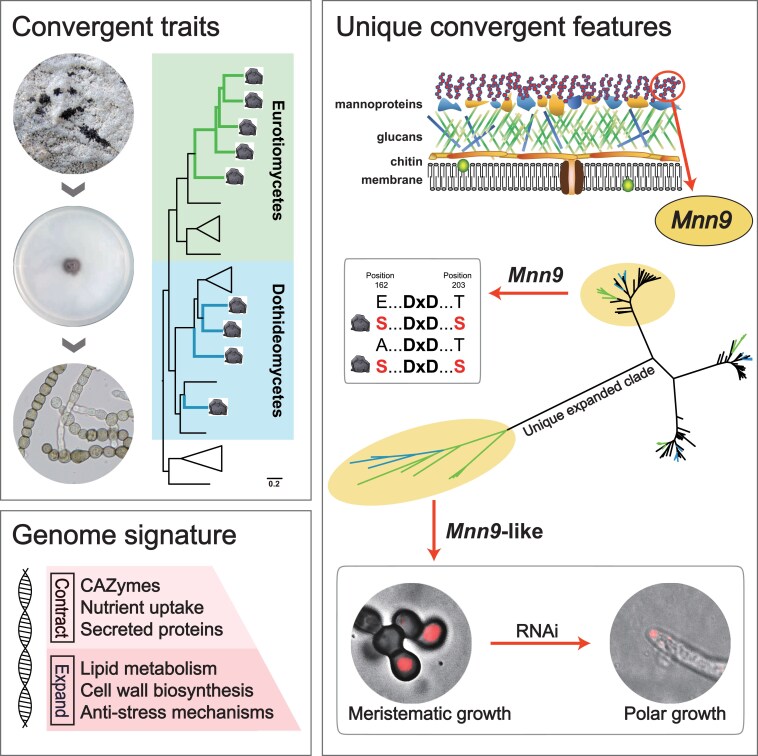

## Introduction

Rock-inhabiting fungi (RIF), commonly known as “black meristematic fungi” and “microcolonial fungi,” are a distinctive fungal group thriving exclusively on barren rock surfaces, even inside, distinctly from lichens in not forming a symbiotic relationship with algae (Staley et al. 1982). Molecular phylogeny has unveiled their wide taxonomic diversity, primarily within Dothideomycetes and Eurotiomycetes classes of the Ascomycota phylum (Selbmann et al. 2008; Ruibal et al. 2009; Sun et al. 2020). Although phylogenetically diverse, these fungi exhibit convergent adaptations to similar rocky environment characterized by elevated levels of radiation, extreme temperatures, desiccation, and limited nutrient availability (Friedmann 1980). RIF have independently developed analogous convergent traits both in morphology and physiology, such as extraordinarily slow meristematic growth, high degrees of melanization, and thick cell walls, to confront a spectrum of environmental challenges and exhibit noticeable convergent evolution (Wollenzien et al. 1995; Ruibal et al. 2008).

Due to high environmental constraints exerting selective pressure, RIF prioritize stress tolerance and survival over competitive abilities. Their meristematic growth, a key characteristic, facilitates resistance against extensive evaporation under desiccation and extreme temperatures, owing to the minimal surface-to-volume ratio (Wollenzien et al. 1995). While shifting to meristematic development triggered by environmental extremes is prevalent in extremophilic fungi (Kirchhoff et al. 2019), RIF have adopted this as a stable trait, enabling the formation of slowly expanding, cauliflower-like colonies through isodiametric enlargement (Wollenzien et al. 1997). Stress resistance in RIF is also conferred by melanin, thick cell walls, and, in some species, extracellular polymeric substances (Breitenbach et al. 2018). For instance, RIF developed multilayered cell walls in response to extreme temperatures (Sterflinger 1998). Supporting this, a recent transcriptomic study of the Eurotiomycetes RIF *Cladophialophora brunneola* showed that genes involved in melanized cell wall biogenesis were significantly upregulated under stimulated drought stress (Fu et al. 2023).

Convergent evolution denotes the independent emergence of analogous traits or features in species or lineages that are unrelated or distantly related (Losos 2011). This phenomenon typically arises when these species inhabit similar ecological niches and respond to comparable selective pressures. Positively selected or rapidly evolving genes are often associated with environmental stress and contribute to individual's fitness (Li et al. 2016; Cao et al. 2017; Xia et al. 2017). Investigations of adaptive evolution at molecular level involve identifying alterations in protein sequence (e.g. convergent and parallel amino acid substitutions), driven by positive selection that result in the fixation of specific substitutions conferring a selective advantage (Zhang and Kumar 1997; Liu et al. 2010; Hu et al. 2017). Genetic convergence can also manifest as variations in gene families (Qu et al. 2013), changes in genes within common biological pathways (Berens et al. 2015), shifts in genome composition (Lyu et al. 2018), or even alterations in gene expression (Hao et al. 2019).

In the field of evolutionary biology, the routine sequencing of fungal genomes plays a crucial role in data mining and comprehending adaptive evolution. Although genome sequencing efforts targeting RIF have increased in recent years (Coleine et al. 2017; Teixeira et al. 2017; Ametrano et al. 2019), taxonomic and ecological coverage remains incomplete. *Cryomyces antarcticus* was the first sequenced RIF genome and did not reveal significant differences from related species and mesophilic hyphomycetes, possibly due to its poor assembly quality (Sterflinger et al. 2014). However, unique genomic features linking to meristematic growth and cold adaptation were revealed in *Friedmanniomyces endolithicus* (Coleine et al. 2020). Noteworthy among these was the draft genome sequence of *Knufia petricola* (Tesei et al. 2017), proposed as a model rock-inhabiting organism, aiding in genetic manipulation to elucidate its rock-dwelling lifestyle and stress survival strategies (Noack-Schönmann et al. 2014; Voigt et al. 2020). Additionally, the genome and long-term stress responsive transcriptome of *C. brunneola* uncovered the necessity of activated melanin and lipid metabolism for sustained growth and lithophilic adaptation (Fu et al. 2023). Continued research on various RIF is still required to elucidate their evolution, adaptation, and the mechanisms contributing to success in extreme environments.

In the present study, we extended our genomic exploration by sequencing three additional RIF in Eurotiomycetes and three in Dothideomycetes, collectively representing nearly all major RIF lineages. Comparative genomic analysis was supplemented with the inclusion of three existing RIF genomes and 18 closely related non-RIF species, and our objectives were threefold: (i) to unveil convergent genomic features of RIF compared with non-RIF; (ii) to identify genes exhibiting convergent positive selection and identical amino acid substitutions among RIF species; and (iii) to investigate functions of unique genes or pathways critical in the adaptation of RIF to rocky habitats. This study will shed light on the genomic underpinnings driving the convergent evolution of RIF, which originated from diverse evolutionary lineages.

## Results

### Phylogeny and Genomic Characteristics of RIF

Six typical rock-inhabiting fungal species, including *Anthracina ramose* CGMCC 3.16372, *Bradymyces pullus* CGMCC 3.17288, *Catenulostroma hermanusense* GZ55-1, *Lithophila catenulate* CGMCC 3.14885, *Lapidomyces* sp. CGMCC 3.30117, and *Rachicladosporium* sp. CGMCC 3.30118, were sequenced and de novo assembled. These genomes were assembled into 13-53 scaffolds, with assembly length ranging from 24.6 to 29.4 Mb and predicting a total of 10,000 to 11,761 gene models. Using the Ascomycota database, BUSCO analysis revealed a high level of completeness ranging from 96.5% to 98.9% (Table 1) (Seppey et al. 2019). Previously reported RIF genomes, including *C. brunneola* CGMCC 3.18770 (Fu et al. 2023), *Coniosporium apollinis* CBS 100218 (Teixeira et al. 2017), and *Knufia petricola* MA 5789 (Tesei et al. 2017), were obtained from GenBank to enrich the diversity and taxonomic representation of our genomic dataset. Additionally, eighteen other ascomycetous fungi, encompassing saprophytic, phytopathogenic, entomopathogenic, endophytic, and lichenized forms, were selected for comparative genomic analysis (Table S1).

**Table 1:** Genome assembly statistics of RIF

Species name (stain)	Isolation locate	No. scaffolds	Length of N50 (bp)	Genome size (bp)	Protein number	BUSCO (%)	GC content (%)
*Anthracina ramose* (CGMCC 3.16372)	Henan, Karst landform	23	2,213,505	28,023,014	10,000	96.5	49.39
*Bradymyces pullus* (CGMCC 3.17288)	Gansu, Desert area	55	3,530,694	29,490,499	11,185	97.3	49.59
*Catenulostroma hermanusense* (GZ55-1)	Guizhou, Rocks	53	1,008,637	27,547,763	11,761	98.7	53.18
*Lapidomyces* sp. (CGMCC 3.30117)	Guangxi, Stone relics	13	2,018,184	24,604,082	10,549	98.9	52.42
*Lithophila catenulate* (CGMCC 3.14885)	Beijing, Rocks	42	4,257,975	28,020,065	11,457	97.3	50.06
*Rachicladosporium* sp. (CGMCC 3.30118)	Tibet, Plateau area	52	1,453,977	24,600,606	10,933	97.7	55.64

**Fig. 1.**
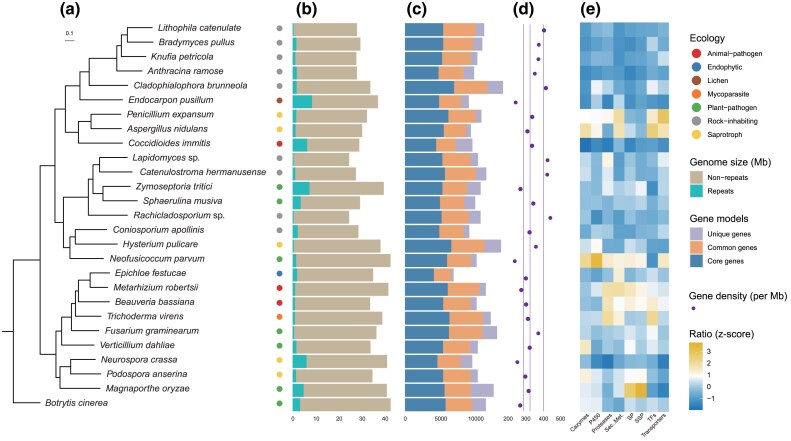
Phylogenetic diversity and genomic overview of RIF within Ascomycota. a) Maximum-likelihood phylogeny of 27 Ascomycota fungi (9 RIF and 18 non-RIF) based on 1,565 single-copy orthologous genes. All bootstrap support values exceeded 90%. b) Genome size composition, showing the proportion of repetitive and nonrepetitive sequences. c) Gene model counts, categorized as core (universal), common (shared by ≥2 species), or unique (species-specific). d) Gene density for each genome. Vertical lines indicate the mean density for non-RIF (left), all fungi (center), and RIF (right). e) Abundance of key functional protein families, normalized by proteome size. CAZymes, carbohydrate-active enzymes; Sec. Met., secondary metabolite biosynthesis clusters; SP, secreted proteins; SSP, small secreted proteins; TFs, transcription factors.

We constructed the species tree using 1,565 single-copy orthologs obtained from the above 27 taxa. Phylogenetic analysis revealed that species clustered corresponding to their respective classes when *Botrytis cinera* as outgroup (Fig. 1a). Five RIF species, including *A. ramose*, *B. pullus*, *L. catenulate*, and *K. petricola* from the family Trichomeriaceae, along with *C. brunneola* from the family Herpotrichiellaceae, clustered together with four non-RIF species in the class Eurotiomycetes. The other four RIF species, comprising *Lapidomyces* sp. and *C. hermanusense* in the family Teratosphaeriaceae of the order Mycosphaerellales, *Rachicladosporium* sp. in the family Cladosporiaceae of the order Cladosporiales, and *C. apollinis*, representing an early diverging species within the subphylum Dothideomycetidae, clustered with four non-RIF species in the class Dothideomycetes. The remaining nine non-RIF species formed a distinct clade within the class Sordariomycetes.

Comparison of primary genomic features revealed that RIF displayed a smaller genome size (mean ± standard deviation = 28.06 ± 2.73 Mb) than non-RIF (36.48 ± 4.58 Mb) via Wilcoxon test (*P* < 0.01) (Figure S1A), as well as lower percentage of repetitive sequences (3.8% ± 1.87% for RIF compared with 7.16% ± 5.94% for non-RIF) (Figure S1C). A similar number of coding genes was observed in RIF and non-RIF, except for the predicted gene counts in *C. brunneola* (14,168), which was the highest among genomes examined. Our findings indicated that RIF possess a more compact genome structure, resulting in a higher gene density (396 ± 39 genes/Mb for RIF compared with 302 ± 46 genes/Mb for non-RIF, *P* < 0.01) (Figure S1D). Notably, among all the species examined in this study, the RIF *Rachicladosporium* sp. exhibited the highest gene density at 444 genes/Mb (Fig. 1d).

### Contraction and Expansion of Functional Gene Families in RIF

**Fig. 2.**
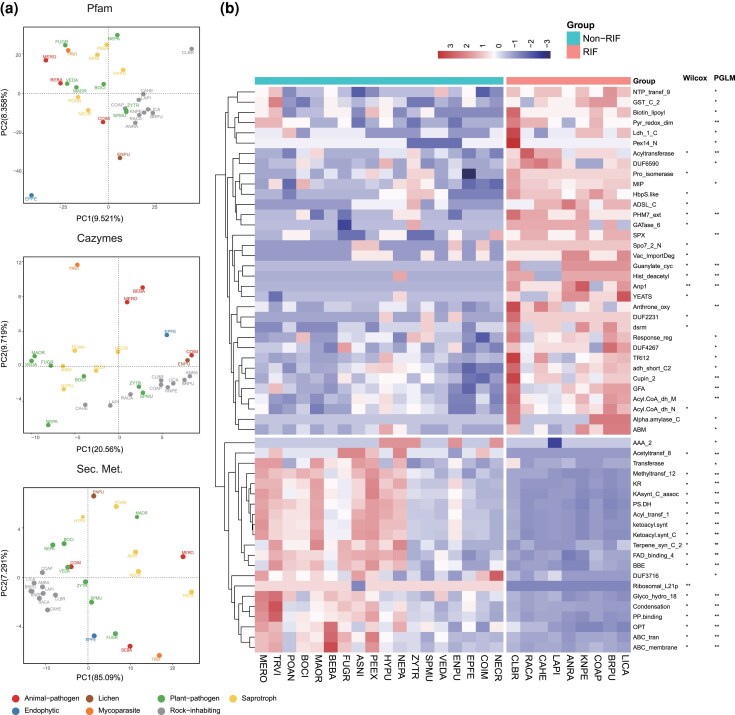
Comparative genomic analysis reveals niche-associated features. a) PCA performed on profiles of Pfam domains, CAZymes, and secondary metabolite synthesis gene clusters (Sec. Met.) clearly separates RIF from non-RIF species. Points represent individual species and are colored by ecological type. Species abbreviations are defined in Table S1. b) Heatmap of Pfam domains with significant abundance differences between RIF and non-RIF, as identified using both a Wilcoxon test and a phylogenetic generalized linear model (PGLM). *, FDR < 0.05, **, FDR < 0.01. Fungal name abbreviations provided in Table S1.

Principal component analysis (PCA) revealed significant disparities between RIF and non-RIF based on the profiles of functional genes, including Pfam, Carbohydrate-active enzymes (CAZymes), and secondary metabolism synthesis gene clusters (Fig. 2a). The genomic heterogeneity was mainly contributed to the contraction of CAZymes (339 ± 74 for RIF compared with 431 ± 123 for non-RIF), secondary metabolites gene biosynthesis clusters (19 ± 8 for RIF compared with 52 ± 16 for non-RIF, *P* < 0.01), secreted proteins (426 ± 113 for RIF vs. 638 ± 244 for non-RIF, *P* < 0.05), and small secreted proteins (118 ± 26 for RIF vs. 227 ± 127 for non-RIF, *P* < 0.05) in RIF (Figure S1E, F, I, and J).

RIF showed a substantial reduction in polyketide synthases, non-ribosomal peptide synthetases, terpene synthases, and indole-producing enzymes (Figure S2A), which correlated with 13 underrepresented Pfam domains: Acetyltransf_8 (PF13523), Methyltransf_12 (PF08242), KR (PF08659), KAsynt_C_assoc (PF16197), PS-DH (PF14765), Acyl_transf_1 (PF00698), ketoacyl-synt (PF00109), Ketoacyl-synt_C (PF02801), Terpene_syn_C_2 (PF19086), FAD_binding_4 (PF01565), BBE (PF08031), Condensation (PF00668), and PP-binding (PF00550) (Fig. 2b;  Table S2). The majority of Polysaccharide Lyases, carbohydrate-binding modules (CBMs), and Auxiliary Activities (AAs) were significantly reduced or absent in RIF (Figure S2B and Table S4). Like AA7, which act as critical oxidative activators that enhance fungal degradation of polysaccharides by activating lytic polysaccharide monooxygenases, reduced in RIF. Particularly, enzymes responsible for chitin and chitosan decomposition, such as chitinases (GH18) and chitin-binding proteins (CBM18 and CBM50), also exhibited a significant decrease in RIF. These reduced enzymes involved in degradation of cellulose, hemicellulose, pectin, and chitin may reflect adaptation of RIF in oligotrophic environments for efficient utilization of complex carbon sources. Transporters containing oligopeptide transporter (PF03169) and ABC transporter (PF00005 and PF00664) also exhibited a remarkable reduction in RIF (Fig. 2b;  Table S2). A smaller proportion of transporters, transcription factors, proteases, and cytochrome P450 monooxygenases (Fig. 1e;  Table S3) in RIF may related to oligotrophic adaptation, distinct from losses in *Coccidioides immitis* (animal pathogenesis) or *Neurospora crassa* (RIP-driven).

Our results revealed a significant expansion of the GT62 family in RIF lineages (phylogenetic regression, *P* = 1.21237E-10, Table S4), which includes mannosyltransferases Mnn9, Anp1, and Van1, critical for synthesizing linear hypermannan in fungal cell walls (Fig. 4b). This finding was further corroborated by a specific analysis of the Anp1 domain (PF03452), which also showed significant expansion in RIF (Fig. 2b;  Table S2). Expanded Ca^2+^-permeable stress-gated cation channel (CSC1, OG0000237) harboring the PHM7_ext domain (Table S3), is likely to cope with drought and osmotic stress (Hou et al. 2014), was observed (Fig. 2b). An additional count of adenylate cyclase (OG0009455, PF00211), responsible for generating the second messenger cAMP (Table S2), suggests that RIF may exhibit enhanced efficiency in signal transdcution and regulatory pathways.

### Positive Selection in Eurotiomycetous and Dothideomycetous RIF

**Fig. 3.**
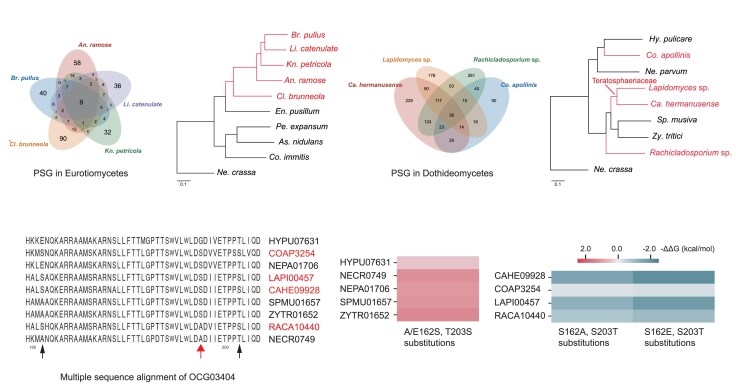
Molecular convergence in RIF. a, b) Venn diagrams of PSGs identified by branch-site model when each (a) eurotiomycetous (*n* = 5) or (b) dothideomycetous (*n* = 4) RIF species (highlighted in red) was designated as the foreground branch in corresponding phylogenetic tree. c) Convergent amino acid substitutions to serine at aligned positions 162 and 203 (black arrows) in the Mnn9 ortholog (OCG03404) across dothideomycetous RIF (highlighted in red). The conserved DXD motif, critical for catalytic activity in glycosyltransferases, is marked by red arrows. d) Predicted changes in protein stability (ΔΔG, kcal/mol) for Mnn9 following amino acid substitutions at positions 162 and 203, calculated using the SPIRED-stab model. Substitutions with RIF-specific residues in five non-RIF species (left) and reverse substitutions in four RIF species (right) are shown. Positive ΔΔG values (red) indicate destabilization, while negative values (blue) indicate stabilization.

To identify positively selected genes (PSGs) shared among RIF adapting to rocky niches, we performed nine independent likelihood ratio tests (LRTs) using a branch-site model separately within Eurotiomycetes and Dothideomycetes, with *N. crassa* as the outgroup. In the Eurotiomycetes analysis of 2,773 single-copy orthologs, we identified 155, 113, 167, 109, and 105 PSGs in *A. ramose*, *B. pullus*, *C. brunneola*, *K. petricola*, and *L. catenulate*, respectively (false discovery rate, FDR < 0.05; Fig. 3a). Nine PSGs (0.32%) were shared among all five eurotiomycetous RIF (Table S5). Similarly, analysis of the Dothideomycetes dataset identified 662, 260, 526, and 674 PSGs based on the 3,290 single-copy orthologs along the branches of *C. hermanusense*, *C. apollinis*, *Lapidomyces* sp., and *Rachicladosporium* sp., respectively (FDR < 0.05; Fig. 3b). Thirty-six PSGs (1.09%) were common to all four dothideomycetous RIF (Table S6). Functional annotation revealed that the majority of these shared PSGs are involved in genetic information processing, including DNA repair and replication (e.g. Endonuclease, DNA replication licensing factor MCM5, Chromatin-remodeling ATPase INO80), transcription (e.g. Transcription regulatory protein SNF2, RNA polymerase RPA2, RPB1, RPB2), and translation (e.g. Pre-mRNA-splicing factors, Pumilio RNA-binding protein family).

Remarkably, two PSGs were shared among all nine RIF, providing compelling evidence of molecular convergency spanning two phylogenetic classes (Tables S5, S6). The first gene, Ino80 ATPase, a chromatin-remodeling complex subunit involved in DNA replication, repair, and transcriptional regulation (Conaway and Conaway 2009; Gerhold and Gasser 2014). The second, ER chaperone BiP, acts as a central regulator of ER stress, facilitating the folding of newly synthesized polypeptides and refolding of misfolded proteins (Gething 1999). The convergent selection of these genes highlights a unified adaptive mechanism: Ino80 maintains DNA stability, while BiP maintains protein homeostasis, together ensuring global cellular stability under extreme environmental challenges.

### Convergent Amino Acid Substitutions in Dothideomycetous RIF

The monophyly of the eurotiomycetous RIF clade (Fig. 3a) limits the power to detect convergent amino acid substitutions, as standard phylogenetic models typically require comparisons across independently evolving lineages. Consequently, we targeted the Dothideomycetes dataset, analyzing three phylogenetic targets for convergence: the Teratosphaeriaceae node (representing the most recent common ancestor (MRCA) of *Lapidomyces* sp. and *C. hermanusense*), the terminal branches of *C. apollinis* and *Rachicladosporium* sp. (Fig. 3b). From an initial set of 3,290 single-copy orthologs, we identified 147 genes with 153 convergent substitutions. To control for random chance, we compared these observations to expectations under the JTT-f_gene_ model via a Poisson test (*q* < 0.05). This procedure filtered 7 genes with 12 highly supported sites, which represent the strongest candidates for adaptive molecular convergence (Table 2).

**Table 2.** Convergent amino acid substitutions in dothideomycetes RIF lineages

Ortholog iD	Position of amino acid substitutions	*P-*value	FDR	Annotation
OCG03404	T203S, A162S, E162S	6.40E-06	0.021071	Mannan polymerase complexes subunit MNN9
OCG02812	V36M	0.00012	0.04372	NADH-ubiquinone oxidoreductase 19.3 kDa subunit, mitochondrial
OCG02418	Q127A, N349D	2.76E-05	0.045483	Synembryn-like protein C3E7.04c
OCG02617	I184T	0.000113	0.046535	Eukaryotic translation initiation factor 3 subunit I
OCG03414	K149R	0.000158	0.047368	\
OCG00628	M406I, G615A	0.000187	0.047403	Probable coatomer subunit gamma
OCG02679	I59L, M62L, S23Q, T23Q	0.000202	0.047463	Endochitinase 3

The most prominent convergent signal corresponds to *Mnn9*, a common subunit of Golgi-localized mannan polymerase complexes I and II (Jungmann and Munro 1998; Kojima et al. 1999). This GT62 family glycosyltransferase, which contains the canonical DxD catalytic motif (Striebeck et al. 2013), acquired two independent serine substitutions (A/E162S and T203S) near its active site (Fig. 3c). Structural modeling predicted that introducing these RIF-specific residues into non-RIF homologs enhances protein stability (negative ΔΔG), whereas reverting them in RIF homologs reduces stability (Fig. 3d). The fact that these stabilizing substitutions occur without altering the secondary structure (Figure S3, S4) supports their potential adaptive significance.

Furthermore, we identified two additional convergent substitutions (M406I and G615A) in the gamma subunit of coatomer complex I, which mediates the retrograde transport of Mnn9 from cis-Golgi to ER for recycling (Todorow et al. 2000; Okamoto et al. 2008; Table 2). Collectively, these results highlight a coordinated adaptive strategy in dothideomycetous RIF that optimizes the stability and trafficking of a key cell wall biosynthesis enzyme.

### A Unique Expanded GT62 Clade Modulates Meristematic Growth in RIF

**Fig. 4.**
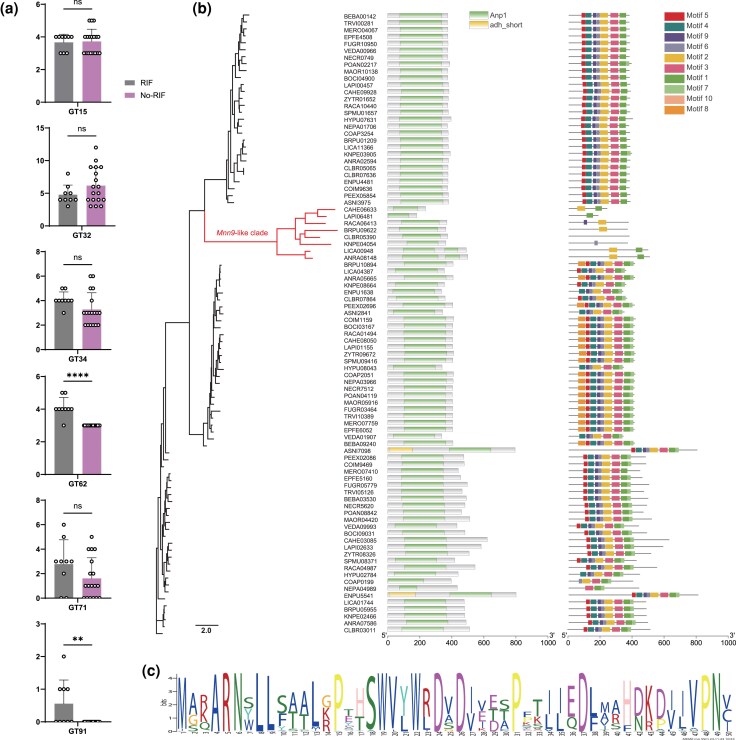
Expansion and diversification of a RIF-specific GT62 clade with mannosyltransferase activity. a) Abundance comparison of CAZymes with mannosyltransferase activity between RIF and non-RIF. Data are presented as mean ± SD; ns, not significant; **, *P* < 0.01, ****, *P* < 0.0001 (two-tailed *t*-test). b) Gene tree of GT62 families from 27 fungal genomes, categorized into *Mnn9*, *Anp1*, and *Van1* clades. A unique, expanded clade (*Mnn9*-like) is exclusively composed of RIF. Taxa are annotated with their corresponding Pfam domains, and conserved motifs are highlighted. c) Consensus sequence of motif 2, which contains the essential DxD catalytic region, across the GT62 families.

Given the adaptive evolution observed in *Mnn9*, we systematically investigated the genomic CAZymes related to cell wall biogenesis (Figure S5). Core enzymes for chitin and β-glucan synthesis (GT2 and GT48 families) were universally conserved across all species (Table S4), indicating their essential roles. As for mannan biosynthesis (Fig. 4a), we identified a novel RIF-specific clade of the GT62 family that was independently expanded in eight RIF species spanning two classes (Fig. 4b). This clade, annotated as *Mnn9*-like based on sequence homology (Table S6), possesses a divergent Anp1 domain with ∼40% similarity to canonical sequences (Figure S6). Notably, this RIF-specific clade exhibits reduced motif conservation, with only motif 2 (DxD catalytic region; Fig. 4c) remaining highly conserved, suggesting potential functional innovation in RIF cell wall biology.

**Fig. 5.**
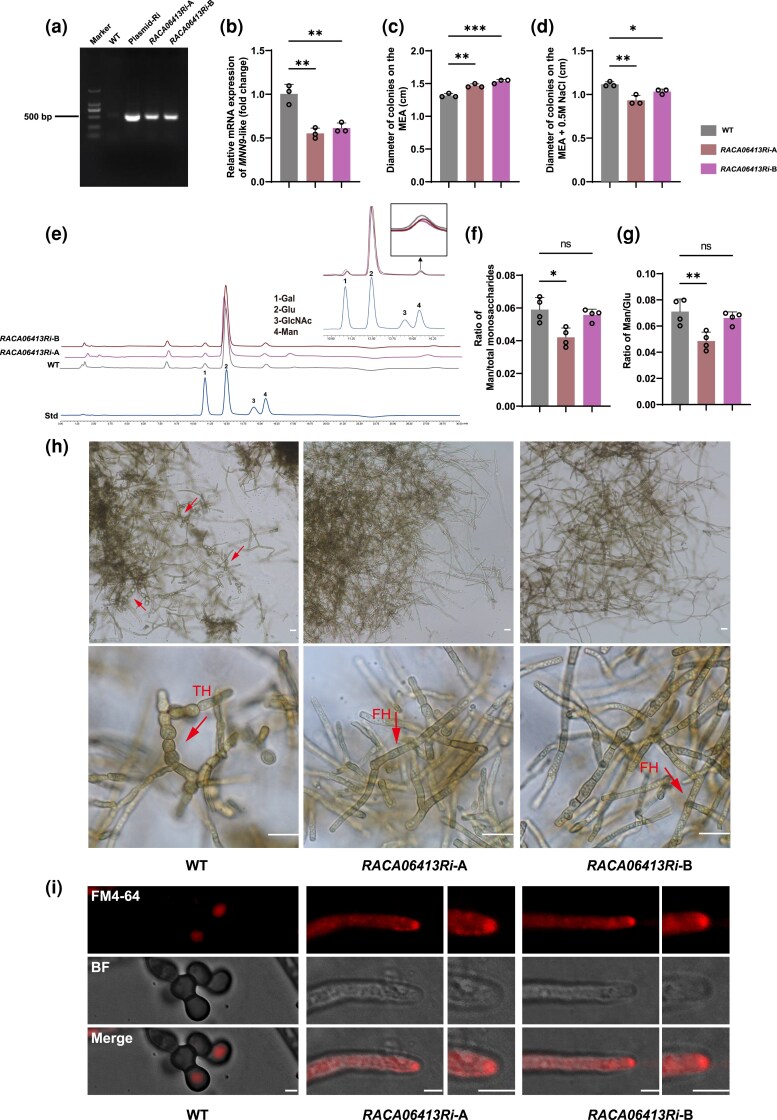
Functional characterization of an expanded *Mnn9* homolog (*RACA06413*) in the dothideomycetous RIF *Rachicladosporium* sp. a) PCR verification of T-DNA insertion using *HPH*-specific primers (HYG-540F/R). A 540-bp amplicon was detected in the RNAi plasmid (positive control) and *RACA06413Ri* strains, but not in the WT. Marker: DNA ladder (150, 250, 500, 750, 1,000, and 1,500 bp). b) Relative expression of *RACA06413* (*Mnn9*-like) in two RNAi strains, showing significant knockdown compared with WT. c) The diameter of colonies was measured in WT and *RACA06413Ri* strains on MEA after 1 month. d) The diameter of colonies was measured in WT and *RACA06413Ri* under osmotic stress (MEA + 0.5 M NaCl) after 1 month. e) HPAEC chromatograms of cell wall monosaccharides from WT and *RACA06413Ri* strains. Peaks: 1, Gal, galactose; 2, Glu, glucose; 3, GlcNAc, N-acetylglucosamine; 4, Man, mannose; Std, monosaccharide standard. f, g) Relative mannose content in *RACA06413Ri*-A, expressed as the ratio to total monosaccharides (f) or to glucose (g). h) Hyphal morphology of WT and *RACA06413Ri* strains. WT exhibits torulose hyphae (TH), while transformants show filamentous hyphae (FH). Scale bar = 10 µm. i) FM4-64 staining Spitzenkörper clustered in hyphal tips. BF, bright field. Scale bar = 2.5 µm. Data are presented as mean ± SD; *, *P* < 0.05, **, *P* < 0.01, ***, *P* < 0.001, ns = not statistically significant (two-tailed *t*-test).

Molecular convergence and lineage-specific expansion of *Mnn9* homologs in RIF suggest enhanced mannan synthesis capacity. To test this hypothesis, an expanded GT62 gene (*RACA06413*) in the dothideomycetous RIF *Rachicladosporium* sp. was targeted for functional characterization. The presence of core RNA-interference (RNAi) machinery genes (Dicer, Argonaute, RdRP; Table S8) confirmed the feasibility of RNAi in this fungus. We constructed an RNAi vector and introduced it via *Agrobacterium tumefaciens*-mediated transformation (ATMT), selecting transformants with hygromycin B (*HPH*) (Figure S7). Genomic integration of the T-DNA was verified by PCR detection of the 540-bp *HPH* fragment (Fig. 5a). Two independent transformants (*RACA06413Ri*-A and *RACA06413Ri*-B) showed significant knockdown of *RACA06413* transcript levels, reduced to 55.3% and 61.4% of wild-type (WT) levels, respectively (Fig. 5b).

Knockdown of *RACA06413* led to distinct phenotypic alterations. The RNAi strains exhibited accelerated growth (Fig. 5c) and increased sensitivity to osmotic stress (0.5 M NaCl) (Fig. 5d). High-performance anion-exchange chromatography (HPAEC) analysis of cell wall polysaccharide in *RACA06413*Ri-A (Fig. 5e) revealed a significant reduction in the mannose-to-total monosaccharide ratio (Fig. 5f, *P* = 0.0112) and mannose-to-glucose ratio (Fig. 5g, *P* = 0.0092). Morphologically, both RNAi strains shifted from torulose hyphae (TH) to filamentous hyphae (FH), indicating a transition from meristematic to polar growth (Fig. 5h, S8-10). This was further supported by the presence of defined hyphal tips with Spitzenkörper, visualized using the fluorescent membrane dye FM4-64 (Fig. 5i). Collectively, these results demonstrate that the expanded *Mnn9*-like clade in RIF is functionally important for mannan synthesis, cell wall integrity, and the characteristic meristematic growth phenotype.

## Discussion

This study reveals common genomic features utilized by RIF, elucidating convergent morphological and physiological traits that facilitate their adaptation to rocky habitats. Generally, RIF possess compact genomes (typically < 30 Mb) with higher gene density, indicating an economical use of genomic resources to accommodate a greater amount of genetic information into limited space and minimize energy consumption (Kelley et al. 2014). Although some RIF species, such as *F. endolithicus* (Dothideomycetes) (Coleine et al. 2020) and *Rachicladosporium antarcticum* (Dothideomycetes) (Coleine et al. 2017), exhibit genome sizes exceeding 40 Mb and encode over 18,000 protein-coding genes, they still display a heightened gene density compared with non-RIF relatives. This pattern may result from environmental stress-induced genome duplication, analogous to adaptations observed in the halophilic fungus *Hortaea werneckii* EXF-2000 (Lenassi et al. 2013).

Nutrient scarcity in rocky environments (Gorbushina 2007) has driven RIF toward an oligotrophic lifestyle, characterized by reduced proportion of secreted proteins, proteases, transporters, and enzymes involved in complex polysaccharides degradation. This nutritional strategy contrasts sharply with those of saprophytic relatives (e.g. *Aspergillus* and *Penicillium* spp.) and phytopathogens (e.g. *Sphaerulina musiva*, *Hysterium pulicare*, and *Magnaporthe oryzae*), which secrete abundant extracellular enzymes to decompose environmental organic matter for nutrient acquisition. In addition to retaining essential melanin biosynthesis capacity, RIF have lost core genes responsible for synthesizing secondary metabolites associated with intercellular communication and defense, reflecting their adaptation to less competitive ecological niches.

While only five Pfam domains and nine orthologs remained significantly enriched in RIF after correction of phylogenetic regression and wilcoxon test (FDR < 0.05), several other gene families showed notable expansion in preliminary screenings. These include overrepresented photolyases (OG0007805; Table S3) for UV damage repair, a higher number of LtrA domain proteins (PF06772; Table S2) linked to low-temperature adaptation in *C. brunneola* (Zheng and Kathariou 1994), and amplified glutathione S-transferases (OG0007434, OG0006966, OG0007960, OG0000179, and OG0007995; Table S3) and HbpS-like proteins (OG0007411 and OG0007286; Table S3), which possibly response to oxidative stress triggered by a cascade of environmental stressors (de Orué Lucana et al. 2009).

To meet the high energy demands of stress tolerance, RIF appear to enhance lipid catabolism (Hapala et al. 2020). Expansions in mitochondrial acyl-CoA dehydrogenases (OG0000758, OG0000838, OG0001327, OG0005123, OG0007022, and OG0007826; Table S3) and peroxisomal acyl-CoA oxidases (OG0000773 and OG0009772; Table S3) suggest enhanced fatty acids β-oxidation. This metabolic adaptation potentially facilitates ATP production and metabolic water generation-a key advantage under desiccation stress (Mellanby 1942), mirroring strategies in fat-rich desert animals. This genomic emphasis on lipid utilization aligns with both the abundant lipid droplets observed in RIF (Gorbushina et al. 2008) and transcriptomic evidence of upregulated of lipid metabolism genes in *C. brunneola* during drought (Fu et al. 2023), strongly supporting a multilevel adaptation in energy storage and mobilization.

Beyond their variation across RIF lineages, PSGs were significantly enriched in genetic information processing pathways, highlighting their essential role in maintaining regular cellular cycles under adverse conditions. Notably, two conserved PSGs, Ino80 ATPase and BiP, exemplify adaptive convergence at distinct regulatory levels: Ino80 modulates chromatin dynamics through sliding nucleosomes, evicting histones, spacing nucleosome on genes, or exchanging histone variants during replication and repair (Clapier et al. 2017), while BiP, a major ER chaperone present in all kingdoms, monitors proteostasis by activating the UPR and preventing protein misfolding (Lee 2005).

The fungal cell wall, a protective matrix, is primarily composed of glucan, chitin, and an outer layer of mannose-rich glycoproteins (Bowman and Free 2006). In RIF, cell wall thickening represents a common adaptation to environmental challenges, supported by convergent evolution in multiple cell wall-related enzymes. For instance, in dothideomycetous RIF, a shared PSG encoding glucan 1,3-β-glucosidase (GH55; Table S6) plays a crucial role in remodeling β-1,3-glucan, a fundamental structural component of the fungal cell wall. Regarding chitin synthesis, the eurotiomycetous RIF *L. catenulate* exhibits the highest number of chitin synthases (CHS), with 18 identified (Figure S5). Specially, two positively selected CHS in dothideomycetous RIF contain an additional *N*-terminal Myosin_head (PF00063) domain, characteristic of class V or VII CHS (Table S6) (Fujiwara et al. 1997; Roncero 2002; Takeshita et al. 2006). This domain not only supports hyphal tip growth and morphogenesis in filamentous fungi (Takeshita et al. 2005; Weber et al. 2006; Larson et al. 2011; Fajardo-Somera et al. 2015), but also enhances tolerance to high temperatures, oxidative stress, and osmotic pressure (Madrid et al. 2003; Liu et al. 2004; Gandía et al. 2014). For chitin remodeling, convergent amino acid substitutions were identified in an endochitinase in dothideomycetous RIF (Table 2). Various proteins with PIR repeats (PF00399) were also abundant in RIF, especially in *L. catenulate* (32 copies) and *K. petricola* (21 copies) (Table S2). These Pir proteins, which are covalently bound to cell wall β-1,3-glucan and undergo extensive *O-*mannosylation (Mrsă et al. 1997), contributing to cell wall integrity and stress resistance (Toh-E et al. 1993). Another noteworthy convergent signal in dothideomycetous RIF was observed in *Mnn9*, a subunit of the mannan polymerase complex (GT62 family) with α-1,6-mannosyltransferase activity involved in N-glycan hypermannosylation. Genomic analyses revealed a distinct expansion of *Mnn9*-like genes in both dothideomycetous and eurotiomycetous RIF, further supporting enhanced cell wall biosynthesis and meristematic growth in the rock-inhabiting *Rachicladosporium* sp. Together, these findings highlight convergent evolutionary patterns in fungal cell wall biosynthesis enzymes, underscoring the structural and adaptive importance of the cell wall for RIF survival in harsh rocky environments.

Mnn9p is a shared subunit of the mannan polymerase complexes M-Pol I and II, which are responsible for biosynthesis of α-core-mannan in *N*-linked glycans (Fabre et al. 2014). The assembly process begins with the initiation of the mannan backbone by Och1p (GT32), followed by primary chain extension catalyzed by M-Pol I, which consists of Mnn9p (GT62) and Van1p (GT62). For further elongation, M-Pol II, composed of Mnn9p, Anp1p (GT62), Mnn10p (GT34), Mnn11p (GT34), and Hoc1p (GT32), progressively appends α-1,6-mannose residues to the main chain (Jungmann and Munro 1998; Jungmann et al. 1999; Kojima et al. 1999). In yeast, this process can extend the outer *N*-glycan chain to over 50 mannose residues (Orlean 2012), with Mnn9p playing a central role in α-1,6-mannosyl chain elongation. Functional studies in *Candida albicans* have demonstrated that deletion of *MNN9* reduces cell wall mannan content by ∼50%, leading to growth defects, impaired hyphal formation, and increased sensitivity to osmotic stress and hygromycin B (Southard et al. 1999). Mannan content and structure also vary considerably across fungal morphotypes. High levels of extensively branched α-1,6-mannan are commonly found in yeast-like species such as *Candida*, *Schizosaccharomyces*, *Citeromyces*, and *Kloeckera* (Ballou 1974; Kobayashi et al. 1987, 1989). In contrast, the filamentous fungus *A. fumigatus* lacks such hypermannosylated structures (Morelle et al. 2005). This pattern is consistent with observations in dimorphic *C. albicans*, where the transition from yeast to hyphal growth is accompanied by a reduction in mannoprotein content and simplification of mannan structure (Machová et al. 2015), underscoring the link between mannan complexity and fungal growth morphology.

In this study, we identified convergent amino acid substitutions in the conserved Mnn9p of dothideomycetous RIF, which were predicted to enhance protein stability without altering its overall structure. RNAi-mediated downregulation of expanded *Mnn9*-like gene in *Rachicladosporium* sp. significantly reduced the mannan content in the cell wall, supporting its involvement in mannan chain biosynthesis. Phenotypic variation between the two transformants (A and B) may reflect differences in RNAi efficiency or the activation of compensatory pathways. Future studies using gene knockout or CRISPR-based approaches will help clarify these functional aspects. Notably, downregulation of *Mnn9-*like triggers a morphological shift from meristematic to polarized hyphal growth, underscoring the intriguing connection between protein mannosylation and growth patterning in RIF. Although functional knockout was not feasible due to limitations in RIF genetic transformation, the reproducible phenotypic changes in transformant A across biological replicates support a role for *Mnn9*-like in cell wall organization and morphogenesis. Changes in cell wall mannosylation are known to influence surface properties such as hydrophobicity, adhesion, and viscosity (Masuoka and Hazen 1997), which may in turn affect cellular morphology and interaction. However, further investigation is needed to determine how the expanded *Mnn9* clade modulates cell wall mannosylation, whether it influences vesicle organization at hyphal tips, and through what specific mechanisms altered mannan levels regulate growth pattern transition and polarity establishment in RIF.

This study reveals shared genetic adaptations among rock-dwelling taxa, though broader taxonomic sampling, particularly in underrepresented lineages such as *Rachicladosporium*, will be essential to confirm their universality. By integrating evidence from genome reduction, gene family evolution, positive selection, and convergent amino acid substitutions, we delineate a comprehensive model of genomic adaptation in RIF. These fungi have evolved compact genomes by shedding both primary and secondary metabolic pathways, while retaining or acquiring genes pivotal for stress resilience. Notably, PSGs were predominantly enriched in genetic information processing pathways, emphasizing the importance of maintaining core cellular functions under environmental extremes. Furthermore, the repeated expansion and molecular convergence of *Mnn9*-like highlight their adaptive relevance, linking enhanced cell wall mannosylation to distinctive meristematic growth habit of RIF. Overall, these findings advance our understanding of how genomic and molecular innovations underpin the success of RIF in some of the most challenging terrestrial environments.

## Materials and Methods

### Fungal Sampling and Isolation

Rock-inhabiting stains, *Anthracina ramosa* CGMCC 3.16372, *B. pullus* CGMCC 3.17288, *C. hermanusense* GZ55-1, *Lapidomyces* sp. CGMCC 3.30117, *L. catenulate* CGMCC 3.14885, *Rachicladosporium* sp. CGMCC 3.30118, were isolated from rock samples collected in Beijing, Henan, Gansu, Guizhou, Guangxi, and the Tibet Plateau in China, respectively (BioProject PRJNA1051547: SAMN38787446–SAMN38787451). Rock fragments (∼0.5–1 cm^3^) exhibiting black colonies were aseptically excised using a stone splitter, surface-disinfected, pulverized, and suspended in physiological saline. The suspension was plated on dichloran rose Bengal chlortetracycline agar supplemented with streptomycin and incubated at 10 °C in darkness for 4 weeks. Slow-growing, dark pigmented colonies subsequently transferred to malt extract agar (MEA) and maintained at 23 °C for over 1 month for purification and identification.

### Genome Sequencing, Assembly, and Annotation

Genomic DNA was extracted from fungal mycelia grown on MEA plates using a cetyltrimethylammonium bromide-based protocol. Sequencing was performed on both the PacBio Sequel and Illumina HiSeq platform at Allwegenes Tech. (Tianjin, China) and Nextomics Biosciences (Wuhan, China), respectively, following the manufacturers’ standard library and sequencing pipeline. Raw PacBio Sequel reads were trimmed, corrected, and assembled using the Canu assembler v1.8 (Koren et al. 2017) with a “correctedErrorRate = 0.035” for error correction and assembly. The assemblies were polished with Pilon v1.23 using Illumina short reads (Walker et al. 2014) to improve base accuracy.

Gene prediction was performed de novo using GeneMark-ES v4.69 (Borodovsky and Lomsadze 2011) and AUGUSTUS v3.4.0 (Stanke and Morgenstern 2005), with RNA-seq data incorporated to refine splice-site annotations. Genome completeness was accessed with BUSCO v5.2.2 (Seppey et al. 2019) against the ascomycota_odb10 database (Table 1). Publicly available fungal genomes and previously sequenced RIF included in this study are listed in Table S1. For consistency, previously annotated genomes retained their original annotations; corresponding protein and coding sequences were retrieved from the NCBI database.

Repetitive elements were identified using RepeatModeler v2.0.3 with RECON v.1.08 and RepeatScout v1.0.5 as core programs to generate a custom repeat library. RepeatMasker v4.0.8 (Chen 2004) was employed to annotate Transposable elements by screening against training library and Repbase (https://www.girinst.org/repbase/) (Bao et al. 2015). Tandem repeats were annotated using Tandem Repeat Finder v.4.04 (Benson 1999).

Functional annotation of predicted gene sets was performed using eggNOGmapper v2.1.9 and InterProScan v5.47-82.0 (Jones et al. 2014) with the default parameters. Secondary metabolite biosynthetic gene clusters were predicted with antiSMASH v7.1.0 (Blin et al. 2019). CAZymes were identified via dbCAN2 (Zhang et al. 2018) using BLASTP v2.10.0 + and HMMER v3.2.1 (Finn et al. 2011) searches.

### Phylogenomic Reconstruction

Orthologous groups were identified using OrthoFinder v2.5.4 (Emms and Kelly 2015; Emms and Kelly 2019). Single-copy orthologs were aligned individually with MAFFT v7.407 in “LISNI” mode (Katoh and Standley 2013), and each resulting alignment was trimmed with Gblocks v0.91b (Castresana 2000). Trimmed alignments were then concatenated into a supermatrix for phylogenetic inference. The best-fitting model was estimated with ModelTest-NG v0.1.7 (Darriba et al. 2020). A maximum-likelihood tree was reconstructed with RAxML v8.2.12 (Stamatakis 2014) under 1,000 bootstrap replicates to assess branch support. Topological consistency was further evaluated with ASTRAL, which showed high gene-tree concordance (quartet score = 0.92). The normalized Robinson–Foulds distance between the concatenation-based and ASTRAL trees was 0.125, indicating strong topological agreement.

### Analysis of Gene Family Expansion and Contraction

To compare gene family, Pfam domain, and Cazymes profile between RIF and non-RIF groups while accounting for phylogenetic relatedness, we applied a phylogenetic regression framework. For Poisson-distributed data (dispersion ≤ 1.5), we used phylogenetic generalized estimating equations (pGEE) implemented in phyloglm with method = “poisson_GEE,” which incorporates Pagel's λ to model phylogenetic covariance. For overdispersed (dispersion > 1.5) or zero-inflated (>50% zeros) data, phylogenetic mixed models were fitted using glmmTMB with a (1|species) random effects. This dual approach ensured that all detected differences represent lineage-independent contrasts after correcting for shared ancestry. All *P*-values were adjusted for multiple testing using the Benjamini–Hochberg FDR procedure, with significance defined as FDR ≤ 0.05.

### Identification of PSGs

The aligned protein-coding genes and the species tree obtained from phylogenetic analysis were used to detect positive selection using the branch-site model in the CodeML of PAML (Yang 1997). Each single-copy gene was analyzed under Model 2 with NSsites = 2. We compared an alternative model (allowing sites under positive selection; fix_omega = 0) against a null model (sites evolving neutrally or under purifying selection; fix_omega = 1 and omega = 1) using LRT. Significance (*P* < 0.05) of the compared LRTs was evaluated by χ^2^ tests from PAML. *P*-values were derived from χ^2^ distributions and further corrected via FDR (Benjamini–Hochberg procedure), with FDR ≤ 0.05 considered significant. Positively selected sites were identified as those with Bayesian empirical Bayes (BEB) posterior probability >95%. To enhance detection sensitivity and minimize background conservation bias, we analyzed nine independent tests, each designating one RIF branch as the foreground, across five Dothideomycetes and four Eurotiomycetes species.

### Detection of Convergent Amino Acid Substitutions

Convergent sites were defined to include both “parallel” and “convergent” substitutions (Zhang and Kumar 1997). Analyses were restricted to single-copy genes. Ancestral sequences were reconstructed using CodeML in PAML v4.9i. Convergent amino acid sites among RIF lineages were identified under two criteria: (i) identical residues across all RIF lineages; (ii) inferred substitution between an extant RIF lineage and its MRCA with non-RIF lineages. To distinguish adaptive convergence from stochastic substitutions, we performed a Poisson test comparing observed convergent sites against expectations under the JTT-f_gene_ model (Zou and Zhang 2015). Benjamini–Hochberg method was applied to correct for multiple comparisons (*q* < 0.05).

### Prediction of Protein Stability Changes

Protein structures were predicted using the AlphaFold server (Abramson et al. 2024). Resulting structures were converted into structure-aware (SA) codes and processed via SaprotHub. Sequence and substitution information were formatted and submitted to SPIRED-Fitness for stability prediction under the SPIRED-Stab model (Chen et al. 2024).

### RNAi Vector Construction, Transformation, and Validation in *Rachicladosporium* sp.

A synthetic construct containing the *Aspergillus nidulans* TrpC promoter, inverted terminal repeats (ITs), an intron flanked by multiple restriction sites, and the *A. nidulans* TrpC terminator was synthesized by GenScript (Nanjing, China). The construct was digested with *Hpa*I and *Nhe*I and cloned into the corresponding site of pAGH3, yielding plasmid pAGH3-R1. A target sequence of *RACA06413* was selected based on whole-genome alignment minimize off-target effects and was amplified from of *Rachicladosporium* sp. cDNA. The resulting amplicon was digested with *Pci*I and *Hpa*I (for upstream insertion relative to the ITs) and with *Nhe*I and *BsrG*I (for downstream insertion), then ligated into pAG1-H3-R1 to generate the final RNAi vector (Figure S7).

The recombinant plasmid was transformed into *A. tumefaciens* strain AGL-1 (Biomed, Beijing, China). An optimized ATMT protocol (Zhang et al. 2003; Zhao et al. 2023) was employed for fungal transformation. *Rachicladosporium* sp. tissues harvested from 25-day-old cultures on corn meal agar (CMA) containing 2 M acetosyringone were cocultivated for 5 days with AGL-1 harboring pAG1-H3-R1. Transformants were selected on MEA supplemented with 100 µg/mL hygromycin B (Leagene, Beijing, China) and 400 µg/mL cefotaxime sodium (Solarbio, Beijing, China). Putative transformants were subcultured onto fresh selection medium, and single-conidial isolates were propagated for five generations on hygromycin B-containing MEA to ensure genetic stability.

Integration of the RNAi construct was confirmed by PCR amplification of a 540-bp fragment of the hygromycin B resistance gene using primers HYG-540F (5′-TTGCAAGACCTGCCTGAAACCGAACTGCCC-3′) and HYG-540R (5′-AACCAAGCTCTGATAGAGTTGGTCAAGACC-3′). Total RNA was extracted from selected transformants and the WT using TRIzol reagent (Invitrogen, Carlsbad, CA, USA) for subsequent transcript analysis.

### Real-time PCR Analysis

RNAs extracted from the WT and RNAi transformants were reverse-transcribed into cDNAs using a FastKing RT Kit with gDNase (TIANGEN, Beijing, China). Quantitative PCR was performed using SYBR Green Real-Time PCR Master Mix (Toyobo, Japan). The *GAPDH* (glyceraldehyde-3-phosphate dehydrogenase) gene was used as an internal control. Relative expression of *RACA06413* was calculated using the 2^−ΔΔCt^ method (Livak and Schmittgen 2001), with three technical replicates per sample.

### Growth and Osmotic Stress Assays

WT and RNAi transformants of *Rachicladosporium* sp. were point-inoculated with 0.5-cm mycelial discs onto MEA with or without 0.5 M NaCl. Colony diameters were measured after 1 month of cultivation at 23 °C.

### Cell Wall Carbohydrate Analysis in *Rachicladosporium*

Fungal strains (WT and RNAi transformants of rock-inhabiting *Rachicladosporium* sp.) were grown in melt extract broth at 23 °C with shaking (180 rpm) for 3 days. Cell walls were isolated as previously described (Fontaine et al. 2000). Briefly, mycelia (10 mg dry weight) were washed, resuspended in 50 mM NH₄HCO₃ buffer (pH 8.0), and disrupted with a homogenizer using 1-mm glass beads at 4 °C. Subsequently, the disrupted mycelial suspension was centrifuged at 8,000 × *g* for 10 min. The pellet was washed three times, treated with 1 M NaOH at 70 °C for 30 min, neutralized to pH 5.0 with acetic acid, and centrifuged. Glycoproteins in the supernatant were ethanol-precipitated (64% final concentration), hydrolyzed in 6 N HCl at 100 °C for 2 h, and dried under vacuum. Monosaccharides were resuspended in 1 mL water.

Samples were purified using a C-18 solid-phase extraction column (Thermo Scientific™ Dionex™), filtered through a 0.22 µm MF-Millipore, and analyzed by HPAEC on a Dionex ICS-5,000+ system equipped with a CarboPac PA20 analytical column (3 × 150 mm) and electrochemical detection. Isocratic elution mode was performed with 2 mM NaOH at 0.4 mL/min and 30 °C. Monosaccharide standards (mannose, glucose, galactose, and N-acetylglucosamine) were processed identically. Peak areas were integrated using Chromeleon 7.2 software (Thermo Fisher), and mannose ratios were calculated after normalization to the glucose internal standard.

### Fluorescence Microscopy

WT and RACA06413Ri transformants hyphae grown on MEA for 7 days at 23 °C were stained with 10 µM FM4-64 for 20 min, washed, and mounted in water. Spitzenkörper were visualized using fluorescence microscope with excitation at 558 nm and the emission at 634 nm.

## Supplementary Material

msaf249_Supplementary_Data

## Data Availability

Raw sequencing reads are available in the Sequence Read Archive (SRA) under BioProject accession number PRJNA1051547. Genome assemblies have been deposited in the Genome Warehouse in National Genomics Data Center (NGDC) under BioProject accession number PRJCA045070. Predicted proteomes from diverse fungal genomes were obtained from publicly available sources, as detailed in Table S1. All other data supporting the findings of this study are available from the corresponding author upon reasonable request. This manuscript does not report original code.
