# Supplementary material for: Genomic Insights Into Convergent Evolution: Adaptation to Rocky Habitats in Rock-Inhabiting Fungi
Source: Mol Biol Evol. 2025 Oct 6;42(10):msaf249. doi: 10.1093/molbev/msaf249 (PMC12560822; doi:10.1093/molbev/msaf249)
Supplement: msaf249_Supplementary_Data [file msaf249_supplementary_data.zip › Final version-Supplemental Figures-Convergent Evol of RIF.docx]

**Genomic Insights into Convergent Evolution: Adaptation to Rocky Habitats in Rock-inhabiting Fungi**

Rong Fu, Luwen Yan, Shunxiang Wang, Dongsheng Wei, Qi Wu*, Xingzhong Liu* and Meichun Xiang*

Corresponding authors: Qi Wu ([wuqi@im.ac.cn](mailto:wuqi@im.ac.cn)), Xingzhong Liu (liuxz@nankai.edu.cn), and Meichun Xiang (xiangmc@im.ac.cn)

**Supplementary Information**

**Supplementary Tables (shown in XLSX file):**

Table S1. Genome information analyzed in this study

Table S2. Pfam domain profiles across RIF and non-RIF species

Table S3. Orthologous gene clusters among RIF and non-RIF

Table S4. Carbohydrate-active enzyme (CAZyme) profiles in RIF and non-RIF

Table S5. Positively selected genes in eurotiomycetous RIF

Table S6. Positively selected genes in dothideomycetous RIF

Table S7. Annotation of the expanded GT62 (*Mnn9*-like) clade in dothideomycetous and eurotiomycetous RIF

Table S8. RNA-interference (RNAi) pathway components in *Rachicladosporium* sp.

**Supplementary Figures:**


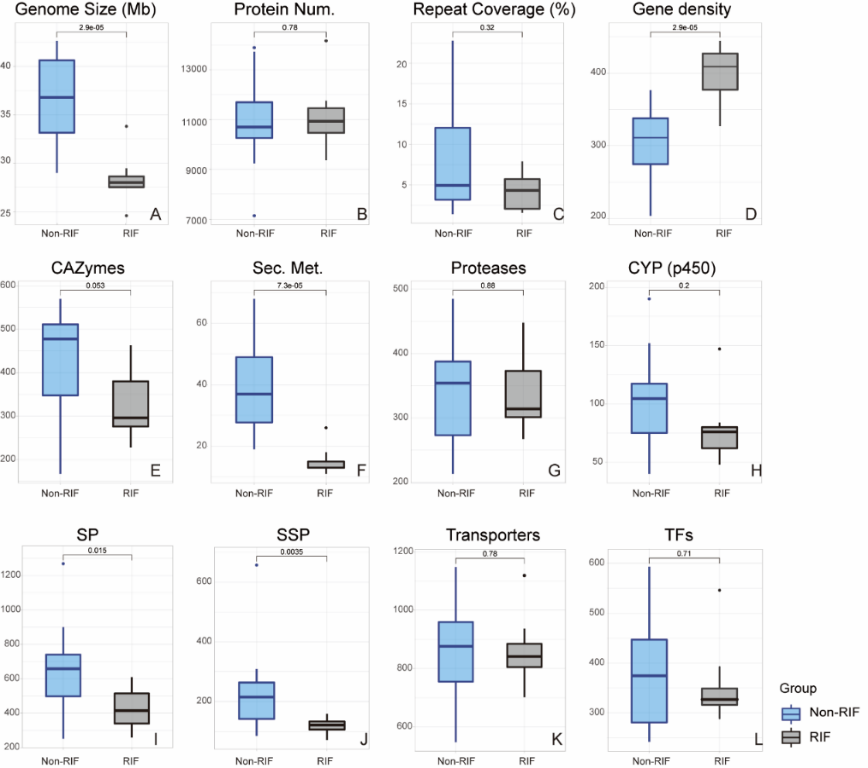


**Figure S1 The Boxplots shows comparison of genomic features and functional genes variation between RIF and Non-RIF via Wilcoxon test**: Genome Size (A), Protein Number (B), percentage of Repeat Coverage (C), Gene Density (D), number of Carbohydrate-Active Enzymes (CAZymes) (E), Secondary Metabolite Synthesis Gene Clusters (Sec. Met.) (F), Protease (G), Cytochrome P450 (H), number of Secreted Proteins (SP) (I), Small Secreted Proteins (SSP) (J), Transporters (K), and Transcription Factors (TFs) (L).


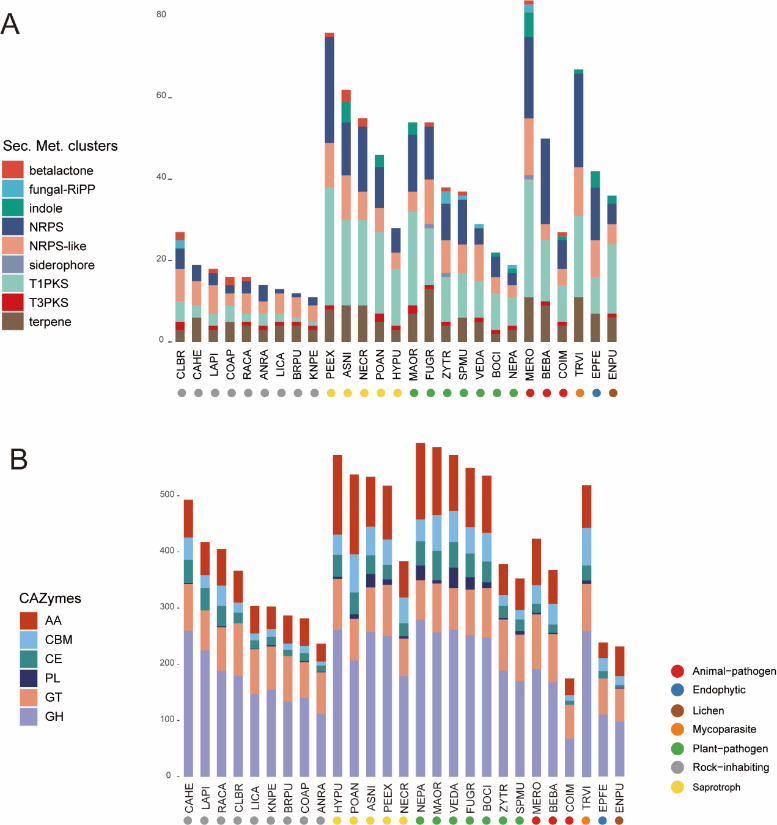


**Figure S2 The barplots shows profiles of secondary metabolite synthesis gene clusters (Sec. Met.) (A) and carbohydrate-active enzymes (CAZymes) (B) among 9 RIF and 18 non-RIF.** Fungal name abbreviations provided in Table S1.


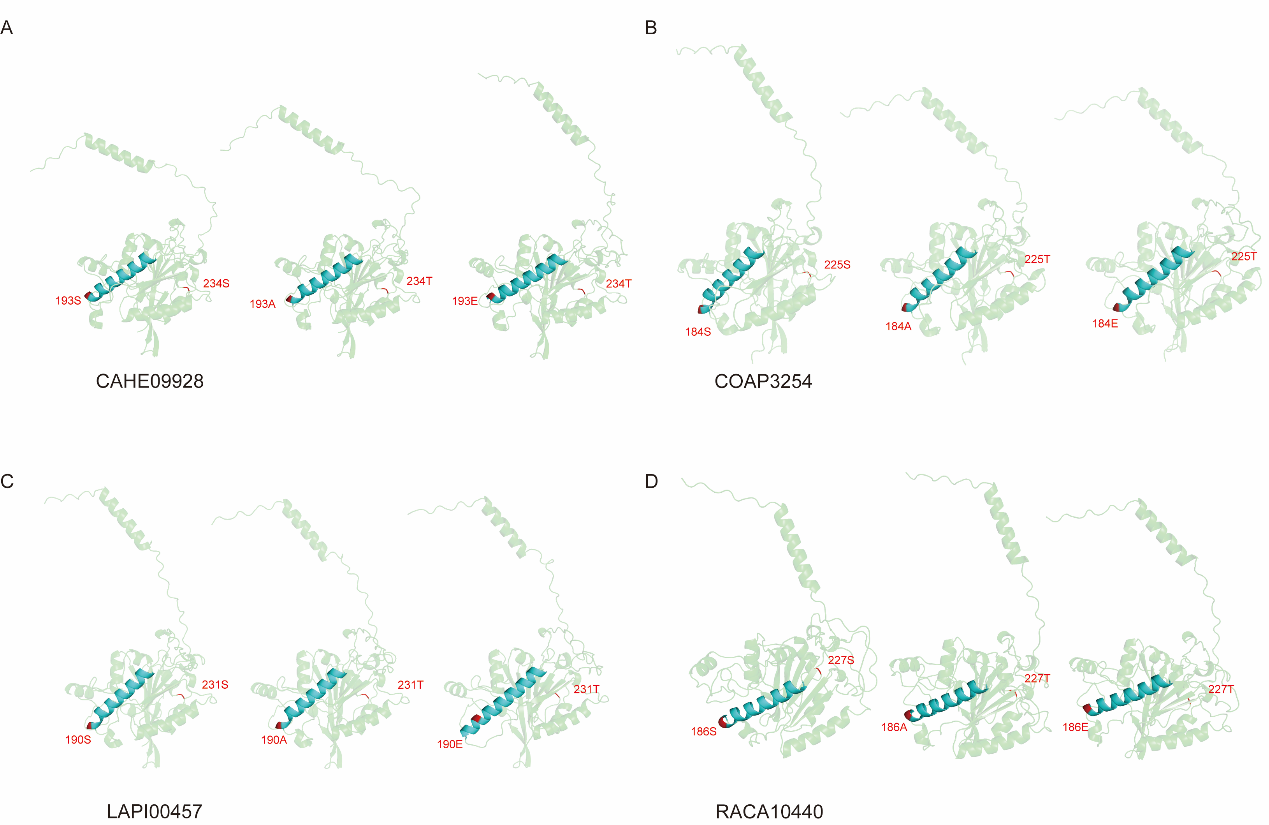


**Figure S3 Structure of Mnn9p following amino acid substitutions at the aligned positions of 162 and 203 in RIF.** (A) CAHE09928 of *Catenulostroma hermanusense*. (B) COAP3254 of *Coniosporium apollinis*. (C) LAPI00457 of *Lapidomyces* sp.. (D) RACA10440 of *Rachicladosporium* sp..


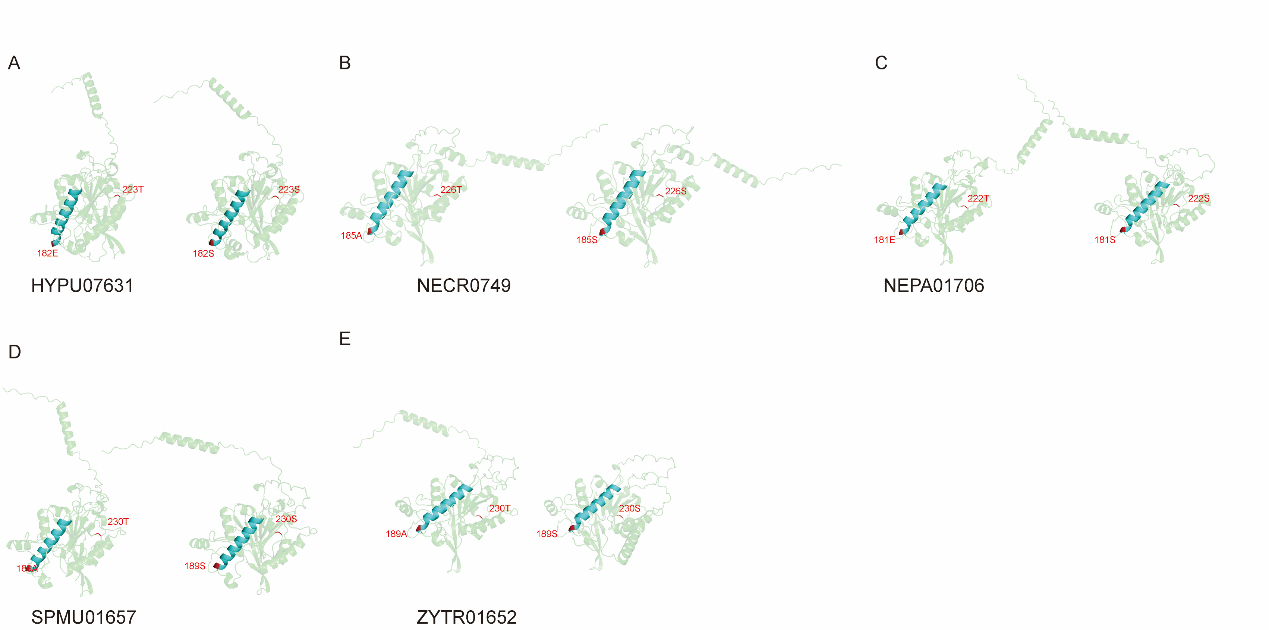


**Figure S4 Structure of Mnn9p following amino acid substitutions at the aligned positions of 162 and 203 in non-RIF.** (A) HYPU07631 of *Hysterium pulicare*. (B) NECR0749 of *Neurospora crassa*. (C) NEPA01706 of *Neofusicoccum parvum*. (D) SPMU01657 of *Sphaerulina musiva*. (E) ZYTR01652 of *Zymoseptoria tritici*.


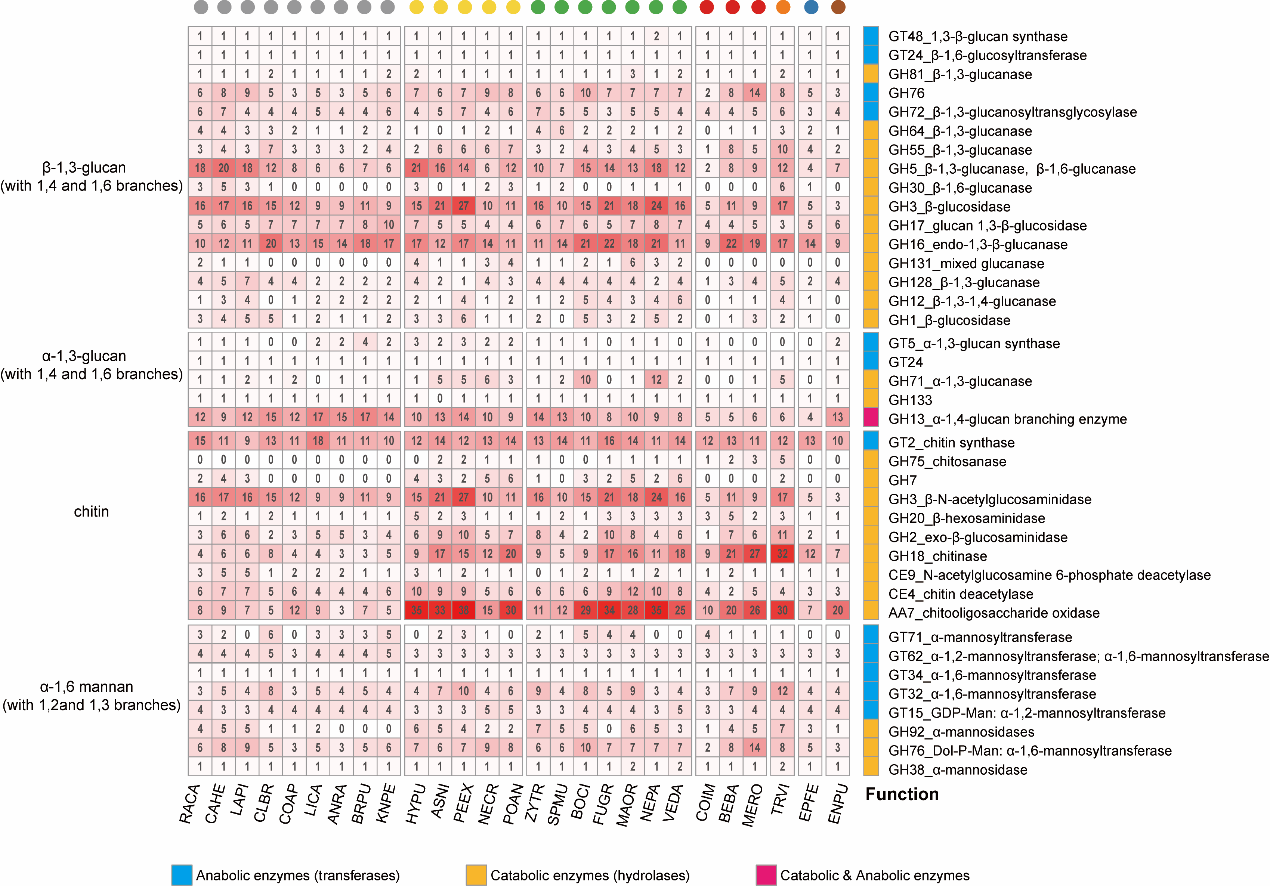


**Figure S5 CAZyme profiles related to fungal cell wall biosynthesis.** Heatmap displaying the abundance of key carbohydrate-active enzyme (CAZymes) families involved in cell wall assembly across the 27 fungal genomes. Species abbreviations correspond to those in Table S1.


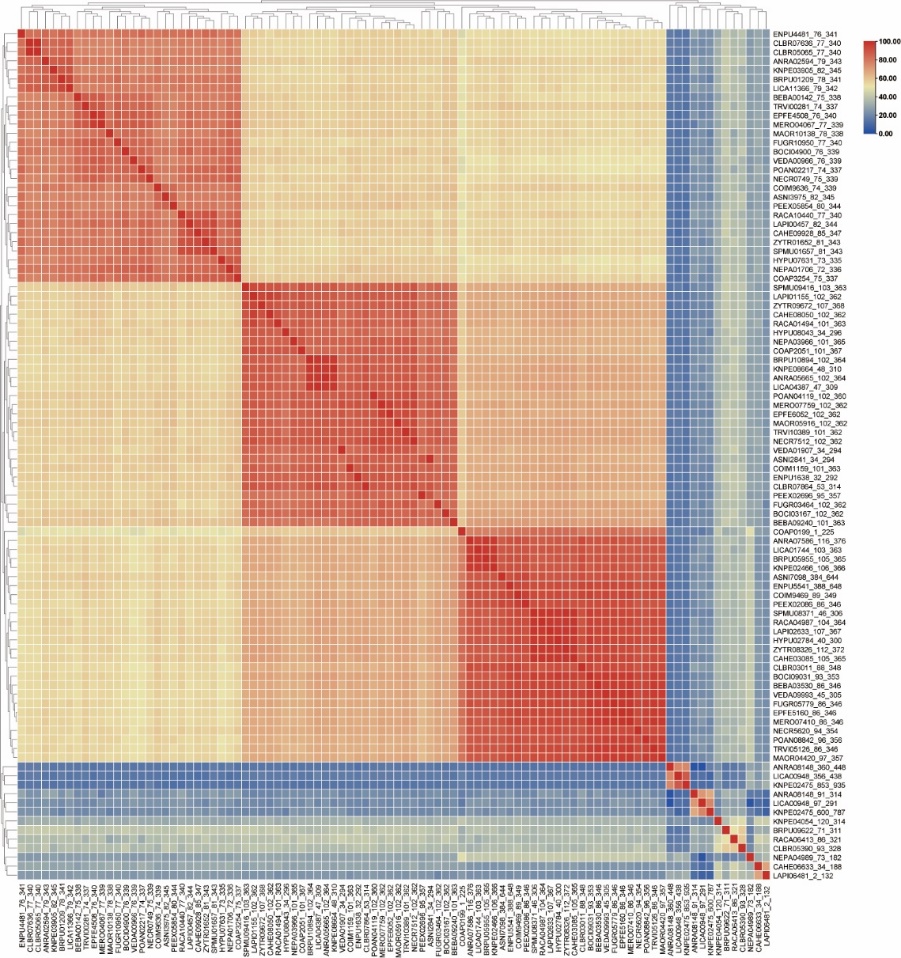


**Figure S6 Sequence divergence of the Anp1 domain within the GT62 family.** Heatmap illustrating the pairwise sequence similarity of the extracted Anp1 domain region from GT62 genes across the 27 fungal genomes. Each sequence is labeled as gene name_domain start_domain end.


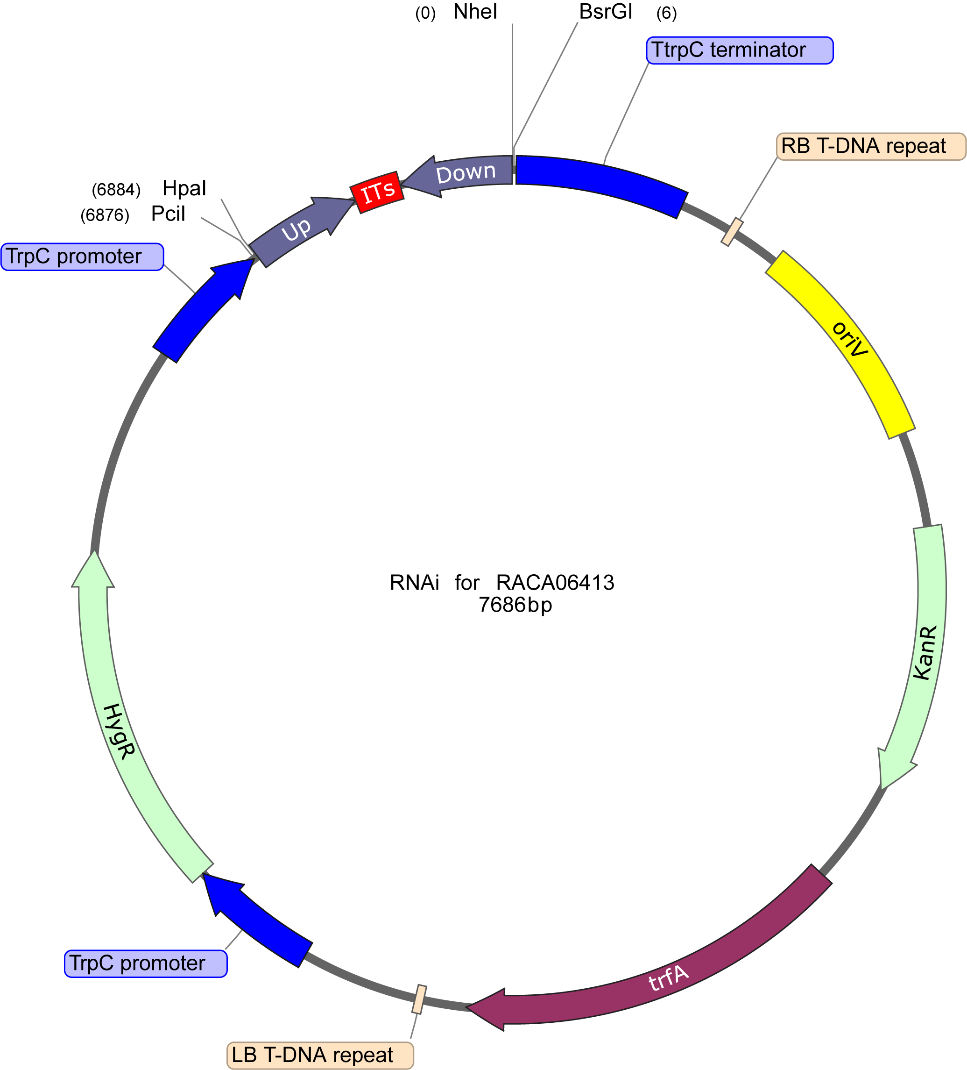


**Figure S7 Schematic of the RNA interference (RNAi) vector used for silencing *RACA06413*.** The construct contains a hygromycin resistance gene (*HPH*) and an inverted repeat sequence targeting *RACA06413*, flanked by T-DNA borders for *Agrobacterium*-mediated transformation.


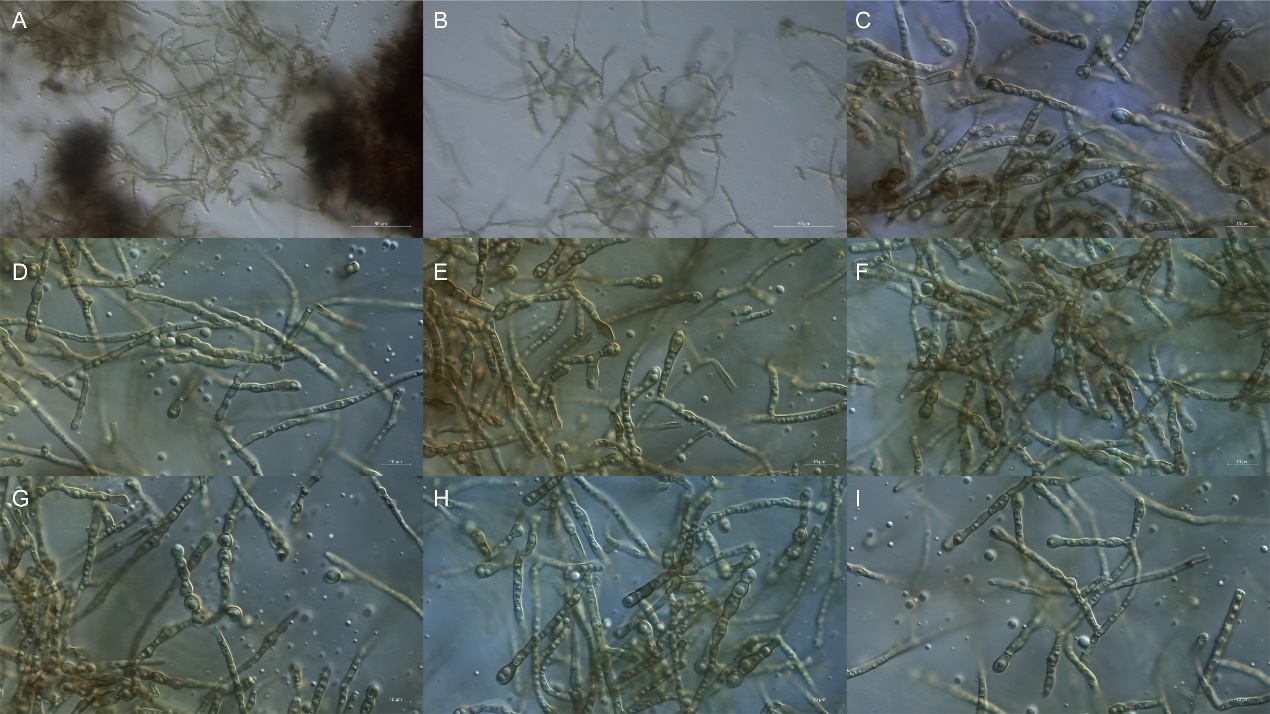


**Figure S8. Hyphal morphology of wild-type *Rachicladosporium* sp.** Representative micrographs from nine randomized fields of view after one month on MEA, showing typical meristematic growth with torulose hyphae. (A, B) Scale bar = 50 µm; (C–I) Scale bar = 10 µm.


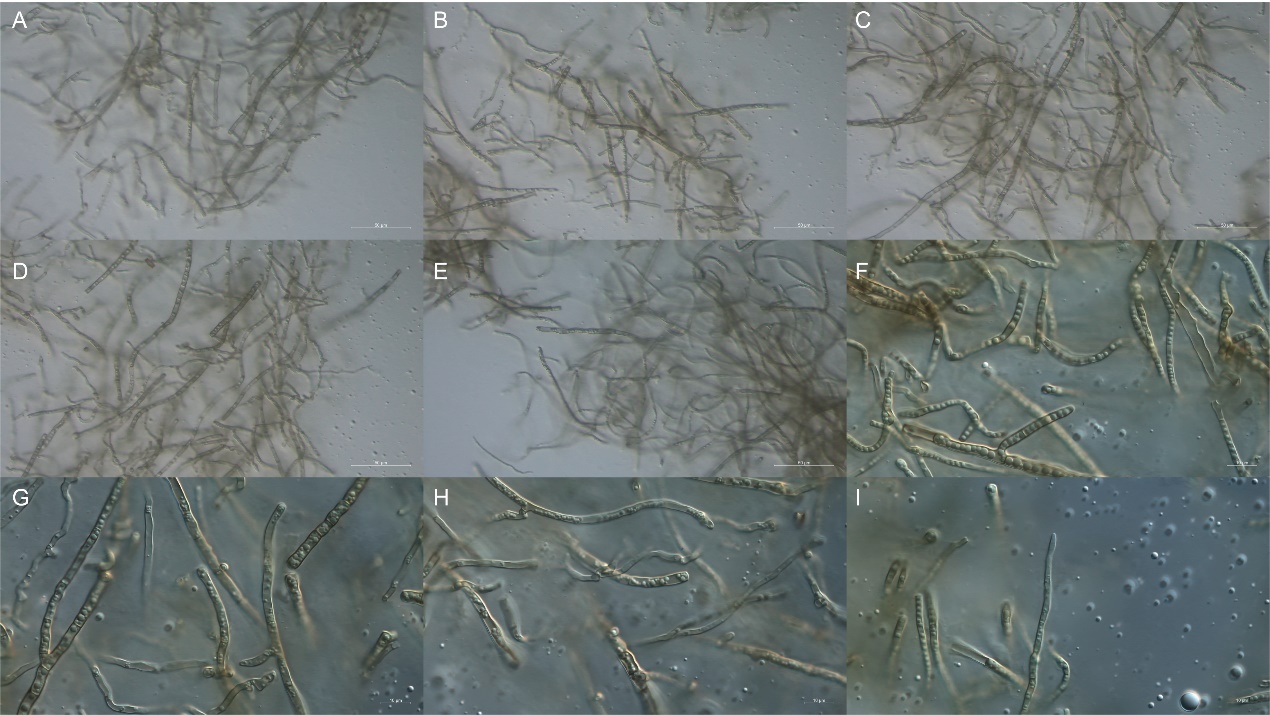


**Figure S9. Hyphal morphology of *RACA06413Ri* transformant A.** Representative micrographs from nine randomized fields confirm the loss of meristematic growth. (A–E) Scale bar = 50 µm; (F–I) Scale bar = 10 µm.


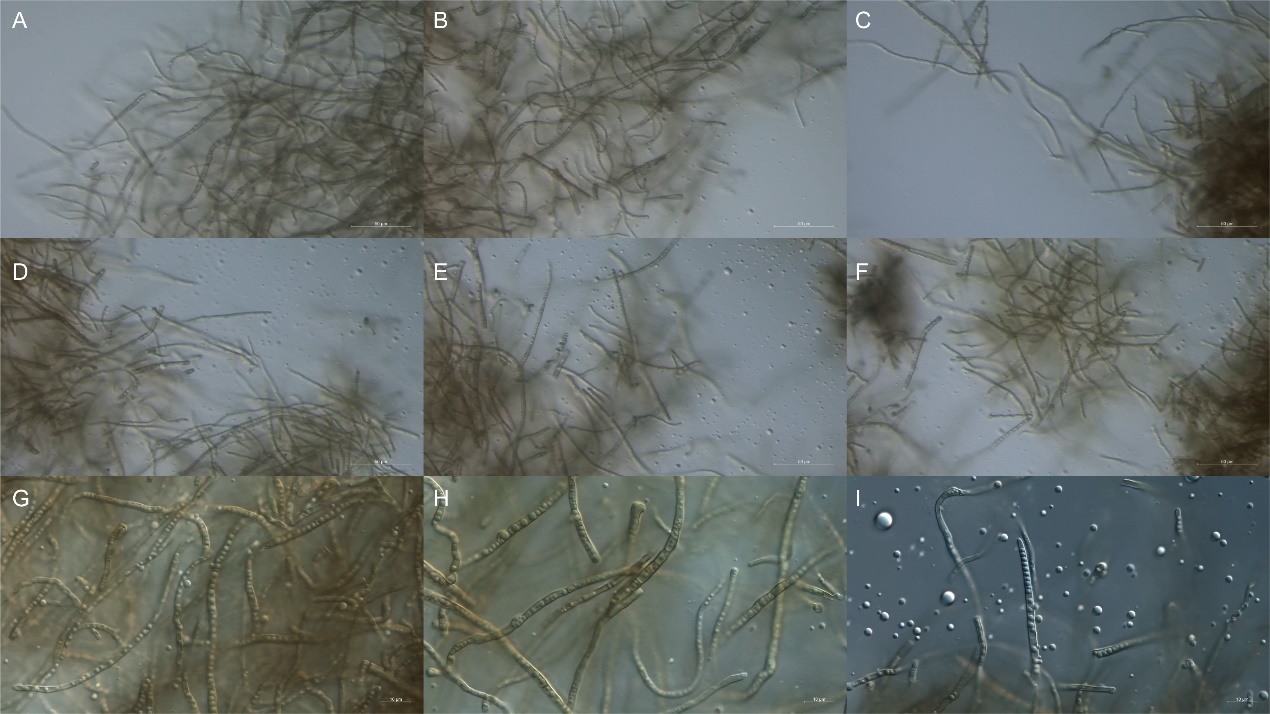


**Figure S10. Figure S10. Hyphal morphology of *RACA06413Ri* transformant B.** Representative micrographs from nine randomized fields confirm the shift to filamentous growth. (A–F) Scale bar = 50 µm; (G–I) Scale bar = 10 µm.
